# Zr-Doped h-BN Monolayer: A High-Sensitivity Atmospheric Pollutant-Monitoring Sensor

**DOI:** 10.3390/s22114103

**Published:** 2022-05-28

**Authors:** Liang-Yan Guo, Sheng-Yuan Xia, Yaxiong Tan, Zhengyong Huang

**Affiliations:** State Key Laboratory of Power Transmission Equipment and System Security and New Technology, School of Electrical Engineering, Chongqing University, Chongqing 400044, China; guoliangyanself@163.com (L.-Y.G.); xiashengyuan@cqu.edu.cn (S.-Y.X.)

**Keywords:** sensor, nanomaterial, Zr-doped h-BN monolayer, simulation and modeling

## Abstract

In the post-epidemic era, industrial production has gradually recovered, and the attendant air pollution problem has attracted much attention. In this study, the Zr-doped h-BN monolayer (Zr-BN) is proposed as a new gas sensor for air pollution. Based on density functional theory (DFT), we calculated and compared the adsorption energies (*E*_ads_), geometric parameters, the shortest distance between gas and substrate (*d*_sub/gas_), density of states (DOS), electron localization function (ELF), charge density difference (CDD), band structure, band gap energy change rate (Δ*E*_g_), and sensitivity (*S*) of Zr-BN adsorption systems (SO_2_F_2_, SOF_2_, SO_2_, NO, and CO_2_ adsorption systems). The results show that Zr-BN had strong adsorption and high sensitivity to the above-mentioned polluted gases, and the sensitivity was in the order of SOF_2_ > SO_2_F_2_ > CO_2_ > SO_2_ > NO. Therefore, this study provides a theoretical basis for the preparation of Zr-BN gas sensors and provides new ideas and methods for the development of other gas sensors.

## 1. Introduction

In the post-epidemic era, rapid industrial recovery has produced large amounts of polluting gases, such as SO_2_F_2_, SOF_2_, SO_2_, NO, and CO_2_ [1,2,3,4,5,6,7,8]. In order to avoid irreversible damage to the environment and human body caused by excessive pollution gases, pollution gases should be controlled and eliminated. To solve this problem, the first step is to monitor the pollution gas in real-time, accurately and intelligently.

Gas-sensing technology is an effective and reliable gas monitoring method [9,10,11]. With the rapid development of material science, two-dimensional nanomaterials, such as graphene, boron nitride, and molybdenum disulfide, have become widely used in gas-sensing technology [12,13,14,15,16,17,18,19,20]. However, due to the physical and chemical inertness of the defect-free monolayer structure, the adsorption between intrinsic boron nitride and small gas molecules is relatively weak, resulting in a relatively poor gas-sensing response [21,22,23,24,25,26,27,28]. As an interesting transition element, Zr has attracted global researchers due to its wide application prospect. Studies have shown that Zr and its complexes can effectively improve the adsorption effect for specific gases [29,30,31,32,33,34,35,36,37]. The gas sensor prepared by it has an appropriate working temperature and good cycle characteristics. At present, atoms or atomic groups are doped on the surface of two-dimensional nanomaterials to improve their gas-sensing response ability mainly through the experimental preparation method and theoretical calculation method. However, the experimental preparation method is time-consuming as well as laborious, and it often causes environmental pollution. The theoretical calculation method based on density functional theory not only has the advantages of being fast, simple, and accurate, and having a low cost, but also can explain the adsorption mechanism of gas-sensing materials from a micro perspective, so as to better modify and optimize the gas-sensing materials [38,39,40,41,42,43,44]. However, there are relatively few studies on gas sensors for the above-mentioned polluted gases.

In this study, an h-BN monolayer, the Zr-doped h-BN monolayer, and five kinds of polluted gases (SO_2_F_2_, SOF_2_, SO_2_, NO, and CO_2_) were constructed based on density functional theory. The adsorption parameters and electronic properties of each adsorption system were calculated, and the adsorption effects of Zr-BN on the above-mentioned polluted gases were explored. The preparation of high-performance Zr-BN gas sensors for atmospheric pollutant monitoring provides a theoretical basis and new ideas and methods for the development of other gas sensors.

## 2. Computational Details

Based on the DFT method, all the models in this study were established and calculated in Materials Studio (MS) software. In order to better describe the non-uniform electron density in the actual system, the Perdew–Burke–Ernzerhof (PBE) functional of the generalized gradient approximation (GGA) was used to deal with the exchange–correlation between electrons. In order to better deal with van der Waals force in the calculation process, all calculations were corrected by DFT-D. In order to simplify the existence of more electrons between atoms in the doping system, DFT semi-core pseudopods (DSPP) were used for simplification using a 6 × 6 × 1 k point for geometric optimization and electronic structure calculation. For the charge transfer between gas molecules and single-molecule layers, the Mulliken population was selected for calculation. In the optimization process, the maximum difference, maximum displacement, and maximum displacement of the iteratively convergent energy were set to 1 × 10^−6^ Ha, 2 × 10^−3^ Ha/Å, and 5 × 10^−3^ Å, respectively. In addition, in order to avoid the intermolecular interaction, the vacuum layer was set to 15 Å. The h-BN monolayer (9 N atoms and 9 B atoms), Zr-BN monolayer, and gas molecular models were optimized in a 3 × 3 × 1 supercell. Considering the influence of a high-humidity environment on the gas sensor, we adopted a higher dielectric constant in Dmol3 solvent in the aqueous solution environment at 298 K, ε = 78.5 C²/(N − M²).

## 3. Results and Discussion

**Figure 1:**
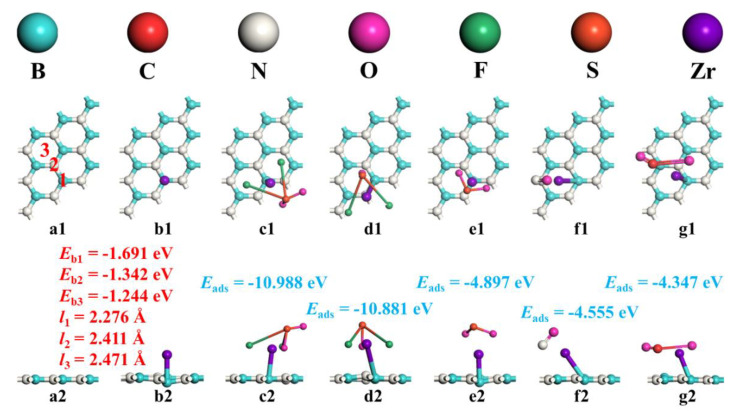
The geometric structures of (**a1**,**a2**) h−BN monolayer, (**b1**,**b2**) Zr−BN monolayer, (**c1**,**c2**) SO_2_F_2_ adsorption system, (**d1**,**d2**) SOF_2_ adsorption system, (**e1**,**e2**) SO_2_ adsorption system, (**f1**,**f2**) NO adsorption system, (**g1**,**g2**) CO_2_ adsorption system.

As shown in Figure 1, the graphical abstraction describes that in an ideal state, a real-time, accurate, and intelligent pollution gas detection system can be established by combining the Internet of Things (IoT) with a Zr-BN gas sensor.

First, h-BN monolayers were constructed, as shown in Figure 1(a1,a2). In order to obtain the most stable Zr-doped h-BN monolayer, one Zr atom was doped over the B atom (position 1), N atom (position 2), and a six-membered ring (position 3) of h-BN monolayer for structural optimization. At the same time, we calculated the geometric structure parameters and formation energy (*E*_b_). In this study, the calculation formula of formation energy was as follows:

*E*_b_ = *E*_Zr-BN_ − *E*_h-BN_ − *E*_Zr_ (1)

where *E*_Zr-BN_, *E*_h-BN_, and *E*_Zr_ represent the energy of the Zr-BN monolayer, h-BN monolayer, and one Zr atom, respectively.

After calculation, the bond lengths between the Zr atom and the substrate formed after doping at three positions were 2.276 Å, 2.411 Å, and 2.471 Å, respectively. The binding energies formed at the three positions were −1.671 eV, −1.671 eV, and −1.671 eV, respectively. This shows that the doping reactions at the three positions were exothermic, which was conducive to the spontaneous formation of Zr-BN, and its stability was position 1 > position 2 > position 3. Therefore, subsequent studies were based on the Zr-atom-doped Zr-BN above the B atom of the h-BN monolayer, as shown in Figure 1(b1,b2).

In order to reasonably explore the adsorption of SO_2_F_2_, SOF_2_, SO_2_, NO, and CO_2_ gas molecules on the Zr-BN monolayer, the gas molecules were placed near the Zr-BN monolayer in different orientations and positions. The most stable adsorption configuration was obtained by optimizing the adsorption structure, as shown in Figure 1(c1–g2). At the same time, the charge transfer (Δ*Q*), the shortest distance between gas and substrate (*d*_sub/gas_), and the adsorption energy were calculated. The adsorption energy of this study was calculated as follows:

*E*_ads_ = *E*_gas/Zr-BN_ − *E_h-BN_* − *E*_gas_ (2)

where *E*_gas/Zr-BN_, *E_h-BN_*, and *E*_gas_ represent the total energy of every adsorption systems, the energy of the Zr-BN monolayer, and the energy of the gas molecule, respectively.

The results show that the Zr-BN monolayer had a strong adsorption effect on the above gases, and the adsorption effect was SO_2_F_2_ > SOF_2_ > SO_2_ > NO > CO_2_. The shortest distance between the gas and substrate (*d*_sub/SO2F2_ = 1.942 Å, *d*_sub/SOF2_ = 1.953 Å, *d*_sub/SO_2__ = 2.044 Å, *d*_sub/NO_ = 2.121 Å, *d*_sub/CO_2__ = 1.787 Å) and the amount of transferred charge were similar to the order of adsorption [45,46]. The negative adsorption energy indicates that the adsorption reaction between the Zr-BN monolayer and the above gases can be carried out spontaneously. It is generally believed that the adsorption reaction can be judged as chemical adsorption when the adsorption energy is less than −0.6 eV [47,48]. However, combined with our subsequent research, it was judged that the adsorption systems were between physical and chemical processes. This is very useful for adsorption and desorption between gas sensors and target gases. It provides a fundamental guarantee for the repeated use and cycle performance of gas sensors.

**Figure 2:**
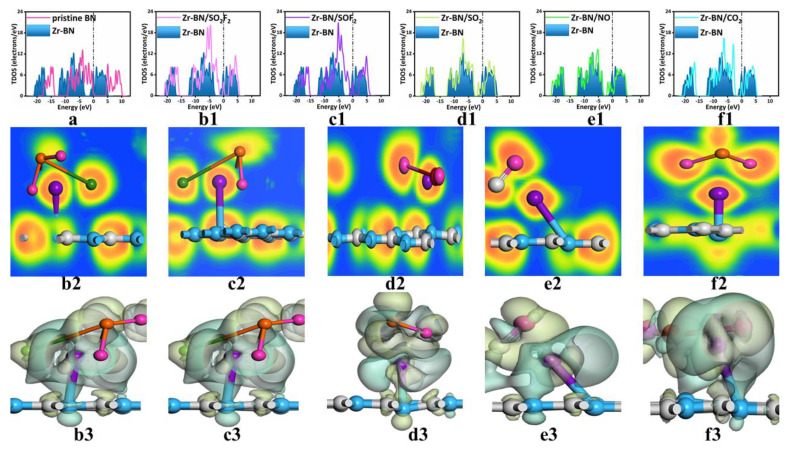
(**a**) The TDOS of h−BN monolayer and Zr−BN monolayer. The TDOS, ELF, and CDD of (**b1**–**b3**) SO_2_F_2_ adsorption system, (**c1**–**c3**) SOF_2_ adsorption system, (**d1**–**d3**) SO_2_ adsorption system, (**e1**–**e3**) NO adsorption system, (**f1**–**f3**) CO_2_ adsorption system. The Fermi energy was set at zero.

In order to further explore the electrical properties of the Zr-BN monolayer and various adsorption systems, we calculated TDOS, ELF, and CDD, as seen in Figure 2. In TDOS, the peak change near the Fermi level had the most important influence on the gas-sensing parameters. The increase or decrease in TDOS far above the Fermi level was far less significant than the change near the Fermi level in the conductivity. This is because the increase in electron filling probability at the highest Fermi level contributes little to conductivity. Figure 2a shows that TDOS significantly shifted to the left after doping one Zr atom, which means that the Zr-BN monolayer was more stable. Continuous TDOS showed that Zr-BN had good conductivity. The TDOS near the Fermi level showed that the doping of one Zr atom significantly improved the electron transition ability of the substrate, which is the key to improving the gas-sensing ability of the substrate. As seen in Figure 2(b1–f1), after the adsorption of various gas molecules, the TDOS of the Zr-BN monolayer changed significantly. One of the common characteristics was seen in that the adsorbed TDOS changed dramatically and decreased near the Fermi level. For the first two adsorption systems, the TDOS changes in the SO_2_F_2_ and SOF_2_ adsorption systems were particularly obvious. This is also consistent with the above two adsorption systems. This is because the doping of Zr atoms improves the molecular structure of the original boron nitride, so the adsorption of gas molecules at different sites will have a moderate impact on the crystal structure. Although the TDOS of the last three adsorption systems (SO_2_, NO, CO_2_) also changed dramatically, it was milder than in the first two adsorption systems. This is because the latter three adsorption systems had a smaller number of carriers than the former two systems. This guarantees that the mixed gas can be detected in real time, accurately, and distinguished macroscopically. At the same time, when the Fermi level was high, the above adsorption systems also underwent great changes, and the common point moved to the left, that is, this adsorption process occurred stably. As shown in Figure 2(b2–f3), when the ELF and CDD were combined, we can see that electrons underwent dramatic changes in the adsorption process. At the same time, the electronic regions in ELFs tended to be fused, which not only shows the strong adsorption between the Zr atom and the adsorbed gas but also shows the adsorption in the five adsorption systems was not chemical adsorption. This proved that the adsorption systems were between physical adsorption and chemical adsorption. In the CDDs of each adsorption system, the electron dissipation area and electron concentration area were extremely dense. This shows that in the process of adsorption, electrons move violently between the substrate and the gas. This also explains why the five adsorption systems of TDOS experience more intense changes before and after adsorption. From the perspective of TDOS, ELFs, and CDDs, Zr-BN can efficiently monitor SO_2_F_2_, SOF_2_, SO_2_, NO, and CO_2_.

**Figure 3:**
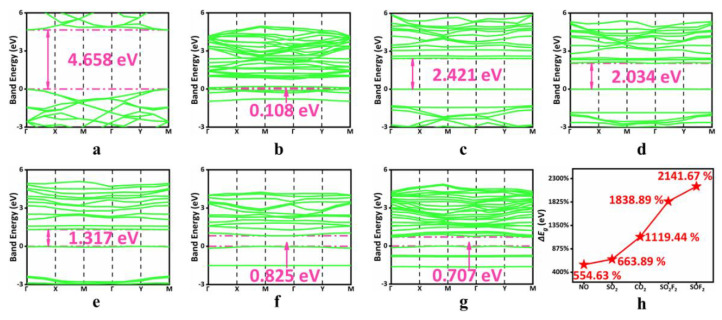
The band energy of (**a**) h-BN monolayer, (**b**) Zr-BN monolayer, (**c**) SOF_2_ adsorption system, (**d**) SO_2_F_2_ adsorption system, (**e**) CO_2_ adsorption system, (**f**) SO_2_ adsorption system, (**g**) NO adsorption system. (**h**) Δ*E*_g_ of all adsorption systems.

In order to further explore the adsorption mechanism, the band structures of h-BN, Zr-BN, and adsorption systems were calculated, as shown in Figure 3. The results show that compared with h-BN, the band gap energy of the Zr-BN monolayer formed by one Zr atom doping above the B atom of the h-BN monolayer was significantly reduced, and the electron transition was easier. After the adsorption of each gas, the band gap energy increased significantly. This means that after the adsorption of gas, the conductivity of the adsorption system decreases, the resistance increases, and either the reaction’s voltage or current macroscopically increases or decreases, respectively. As shown in Figure 3h, the band gap energy change rate changed by 554.63~2141.67%. This is obvious enough to distinguish between the types of adsorbed gases.

In order to further explore the practicability of Zr-BN, the sensitivity (*S*) of each system was calculated. The sensitivity calculation formula used in this study is as follows:

*S* = (1/*σ*_Zr-BN/gas_ − 1/*σ*_Zr-BN_)/(1/*σ*_Zr-BN_) (3)

where *σ*_Zr-BN/gas_ and *σ*_Zr-BN_, respectively, represent conductivity of gas adsorption systems and the Zr-BN monolayer.

**Figure 4:**
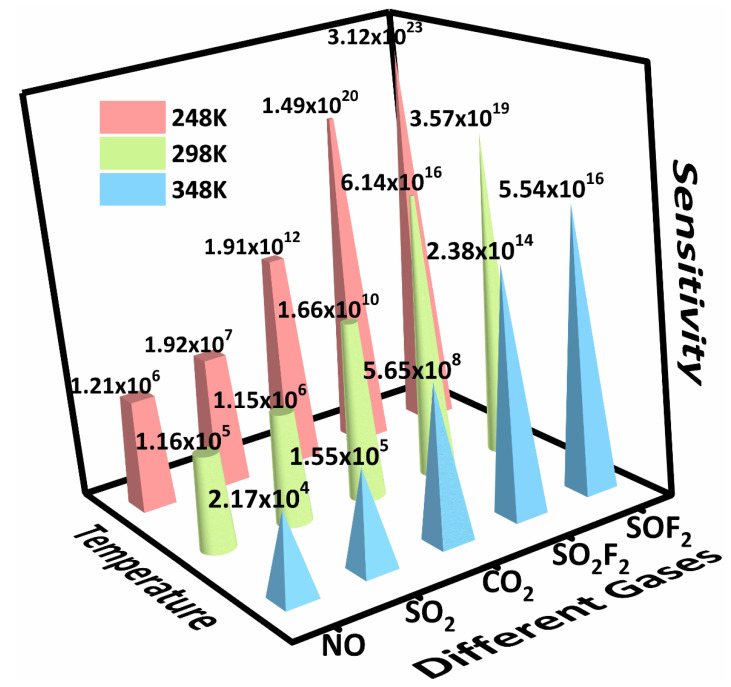
The sensitivity (*S*) of all adsorption systems.

As shown in Figure 4, each adsorption system showed very high sensitivity at different temperatures; the sensitivity was in the order of SOF_2_ > SO_2_F_2_ > CO_2_ > SO_2_ > NO. At room temperature (298 K), the sensitivity of SOF_2_ can reach 3.57 × 10^19^. This fully demonstrates that Zr-BN has great potential to become a gas sensor for monitoring the above-mentioned polluted gases.

## 4. Conclusions

Based on the DFT method, this paper constructed the most stable Zr-BN monolayer and its optimal adsorption configurations with SO_2_F_2_, SOF_2_, SO_2_, NO, and CO_2_, and calculated the adsorption parameters of each adsorption system. By comparing and analyzing the above parameters, the following conclusions were obtained:

(1) The Zr-atom-doped h-BN monolayer is the best doping site above the B atom of the h-BN monolayer.
(2) The adsorption between Zr-BN monolayer and the above gases occurs between physical adsorption and chemical adsorption. The adsorption order is SO_2_F_2_ > SOF_2_ > SO_2_ > NO > CO_2_.
(3) The sensitivity of each adsorption system is high, and the sensitivity is in the order of SOF_2_ > SO_2_F_2_ > CO_2_ > SO_2_ > NO.
(4) The Zr-BN monolayer has the potential to monitor the above-mentioned pollution gases. This paper provides new ideas and methods for the development of other gas sensors.

## Data Availability

Not applicable.
